# Development of a Small-Footprint 50 MHz Linear Array: Fabrication and Micro-Ultrasound Imaging Demonstration

**DOI:** 10.3390/s24061847

**Published:** 2024-03-13

**Authors:** Carlos-Felipe Roa, Emmanuel Chérin, Nidhi Singh, Jianhua Yin, Aaron Boyes, F. Stuart Foster, Christine E. M. Demore

**Affiliations:** 1 Department of Medical Biophysics, University of Toronto, Toronto, ON M5G 1L7, Canada; nidhi.singh@mail.utoronto.ca (N.S.); stuart.foster@utoronto.ca (F.S.F.); 2 Sunnybrook Research Institute, Toronto, ON M4N 3M5, Canada; emmanuel.cherin@sri.utoronto.ca (E.C.);

**Keywords:** high-frequency ultrasound, transducer array, fine pitch, flexible-circuit cabling, electrical characterization, acoustic characterization, miniaturization, polyimide, electrode patterning, laser machining, excimer laser, metal deposition, high-density interconnect

## Abstract

Compact high-frequency arrays are of interest for clinical and preclinical applications in which a small-footprint or endoscopic device is needed to reach the target anatomy. However, the fabrication of compact arrays entails the connection of several dozens of small elements to the imaging system through a combination of flexible printed circuit boards at the array end and micro-coaxial cabling to the imaging system. The methods currently used, such as wire bonding, conductive adhesives, or a dry connection to a flexible circuit, considerably increase the array footprint. Here, we propose an interconnection method that uses vacuum-deposited metals, laser patterning, and electroplating to achieve a right-angle, compact, reliable connection between array elements and flexible-circuit traces. The array elements are thickened at the edges using patterned copper traces, which increases their cross-sectional area and facilitates the connection. We fabricated a 2.3 mm by 1.7 mm, 64-element linear array with elements at a 36 μm pitch connected to a 4 cm long flexible circuit, where the interconnect adds only 100 μm to each side of the array. Pulse-echo measurements yielded an average center frequency of 55 MHz and a −6 dB bandwidth of 41%. We measured an imaging resolution of 35 μm in the axial direction and 114 μm in the lateral direction and demonstrated the ex vivo imaging of porcine esophageal tissue and the in vivo imaging of avian embryonic vasculature.

## 1. Introduction

Recent advances in high-frequency ultrasound (US) imaging have led to applications in preclinical research for imaging small animals and clinical imaging applications in dermatology, cardiology, and ophthalmology [1]. Array-based micro-ultrasound transducers (micro-US: 15–70 MHz operating frequencies) provide excellent soft tissue contrast, real-time imaging capabilities, and resolutions of less than 50 μm [2]. A major drawback of high-frequency imaging is the reduced imaging depth due to increased acoustic attenuation, which can be overcome by positioning the array close to the tissue of interest. The inherent scaling of the transducer dimensions with the wavelength makes high-frequency imaging transducers suitable to be miniaturized for applications that require imaging depths of less than 2 cm, such as gastrointestinal, oral, brain, cardiac, and vascular imaging and preclinical small animal imaging.

The fabrication of high-frequency arrays is challenging and involves trade-offs in material selection, processing methods, array dimensions, and interconnection approaches. Imaging arrays can have several tens to hundreds of elements, each one approximately one wavelength in width (30 μm at 50 MHz), that, generally, need to be addressed individually to enable electronic focusing and beamforming. Handheld probes use an interposer circuit through which individual elements are connected to a long micro-coaxial cable bundle for connection to the beamforming system [3,4,5]. For compact or endoscopic applications of micro-US, the size of the interposer and the electrical interconnect between array elements and the interposer become limiting factors in miniaturization. Space-saving approaches to connect the element electrodes to the cabling are required while satisfying flexibility and length requirements for a given application. Relatively superficial clinical applications such as oral and intraoperative brain imaging may need only centimeter-long probes or a small footprint only at the point of contact, while intraluminal applications like gastrointestinal or intracardiac imaging require cabling of a meter or longer. A minimized probe footprint could be useful for preclinical applications such as imaging the internal organs of small-animal models of disease while avoiding artifacts from bone [6] or evaluating tumor growth or treatment response on avian embryonic chorioallantoic membranes (CAMs), where the surface contact area between the probe and the respiratory organ should be minimized [7,8].

One route to minimizing the size of an ultrasound probe is to reduce the number of cables by placing an application-specific integrated circuit (ASIC) next to or underneath the array. Besides multiplexing to reduce the channel count, the ASIC can perform transmit–receive switching, amplification, and beamforming [9,10,11,12,13]. In addition to the complexity and cost of ASIC implementation, there are technical limitations with the implementation of ASICs operating at high frequencies (>30 MHz) [14,15]. Capacitive and piezoelectric micromachined ultrasound transducers (MUTs) can be fabricated at a large scale and can be closely integrated with transmit and receive electronics to achieve small-footprint transducers [16,17,18]. However, there are limited MUTs that operate at high frequencies (>20 MHz) [19,20].

Piezoceramic arrays continue to be standard for high-frequency imaging, mainly due to their high electromechanical coupling and high dielectric coefficients for high sensitivity. Several novel interconnects and connection techniques have been implemented to fabricate compact and endoscopic arrays based on piezoceramic transducers. For example, a rigid glass transmission line interposer was used to connect a 64-element 60 MHz array to a printed circuit board (PCB) in a 2 mm diameter needle for biopsy guidance [21]. The element electrodes, ending in 50 μm diameter bond pads, were connected to the transmission line traces with silver-coated glass spheres and conductive epoxy using a flip-chip bonding process. Flexible printed circuit boards (FPCBs) with traces matching the micro-US array pitch have been used to connect directly to element electrodes, either as a layer between the array and the backing layer [22,23,24,25] or at the end of extended array electrodes using anisotropic conductive adhesives [26], and packaged into needles [22,26] or catheters [23]. However, in these examples, a bend in the flexible circuit is necessary to avoid strain in the traces or in the acoustic stack, adding 0.5 to 1 mm to the elevation dimension. Bezanson et al. showed that the bending radius can be avoided, thereby reducing the probe footprint, by wire bonding between bond pads extending from the array elements to the edges of a multilayer FPCB cut at filled vias [27]. For a 64-element 40 MHz phased array, 700 μm was added to the elevation dimension for a double-sided interconnect, and the device was packaged in a 2.5 × 3.1 mm housing for neurosurgery applications.

To further reduce the array footprint, we propose a method that achieves a right-angle connection between the array element electrodes and the FPCB traces, eliminating the bending radius and adding less than 200 μm in elevation. We use highly flexible FPCBs with traces at twice the array pitch, connected in an interdigitated manner to the element electrodes via vacuum-deposited, laser-patterned, and electroplated metals. We describe the fabrication of a laser-diced 2-2 piezocomposite forward-looking array and the interconnection method. The device is presented with electrical and acoustic characterization. The ex vivo phantom imaging of swine tissue and in vivo imaging of CAM vasculature demonstrate the array functionality.

## 2. Array Design

**Figure 1:**
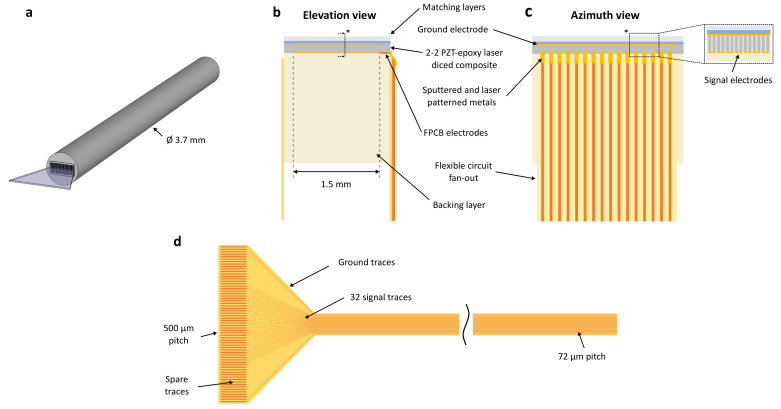
The array transducer design showing (**a**) the design concept with the array fitting in a 11 Fr catheter, (**b**) elevation cross-section and (**c**) azimuth views, and (**d**) the flexible printed circuit board with 72 μm pitch traces for an interdigitated connection to array elements. * Position and orientation of sectional view.

The device comprises a 2.3 by 1.7 mm, 64-element forward-looking array with a 36 μm element pitch, as illustrated in Figure 1. The transducer layers comprise a sub-diced 2-2 piezocomposite with two piezoelectric ceramic pillars per array element and a non-conductive epoxy filler, two quarter-wavelength matching layers, and a non-conductive epoxy backing layer loaded with a dense powder, as displayed in Figure 1b,c. The element pitch (1.2λ) was selected to be slightly larger than the typical one-wavelength guideline to increase the length of the array and the image width.

The final transducer design (Table 1) was determined following the guidelines described in [28,29]. First, an initial set of dimensions and acoustic impedances were calculated based on the KLM (Krimholtz–Leedom–Matthei) equivalent circuit model [30] using PiezoCAD (Sonic Concepts Inc., Bothell, WA, USA) and a target wavelength in water of 30 μm (50 MHz center frequency). Then, simulations using a finite element model (FEM) were performed in OnScale (OnScale Inc., Cupertino, CA, USA) to more accurately model the electric and acoustic performance of the device in two dimensions.

For the active layer, a PZT-5H-type ceramic (3203HD, CTS Corporation, Lisle, IL, USA) was selected for its excellent piezoelectric properties in high-frequency operation [3] and Epo-Tek 301 (Epoxy Technology, Inc., Billerica, MA, USA) as a filler in the laser-diced 6 μm kerfs. The effective material parameters for the 2-2 piezocomposite design were determined for the KLM model using a simplified thickness-mode oscillation model, described in [31,32]. An effective impedance of 23 MRayl was calculated for the piezocomposite with a 67% volume fraction. The element dimensions used in the simulation were 30 μm width with 6 μm kerf, 1500 μm length (elevation), and, initially, 35 μm thickness.

A two-stage matching layer was used with the aim of broadening the frequency response and improving efficiency [33]. The target acoustic impedance of the first and second matching layers, $Z_{ML1}$ and $Z_{ML2}$, respectively, were calculated with

(1) $$\begin{array}{c} Z_{ML1}=Z_{PZT-C}^{4/7}Z_{W}^{3/7}\\ Z_{ML2}=Z_{PZT-C}^{1/7}Z_{W}^{6/7}, \end{array}$$

where $Z_{PZT-C}$ and $Z_{W}$ are the acoustic impedances of the PZT composite and water, respectively, yielding 7.10 and 2.21 MRayl target impedances. The material properties for the epoxy–PZT powder composites utilized for the first matching and backing layers were computed using the model proposed by Devaney and Levine [34,35], which describes the elastic properties of a two-phase composite. The initial matching layer thicknesses were chosen to be equal to one-quarter of a wavelength at 50 MHz. For the first matching layer, a non-conductive epoxy loaded with PZT powder (32% *v*/*v*) was selected for a target impedance of 7.09 MRayl and a thickness of 11 μm. An unloaded epoxy was used for the second matching layer with an impedance slightly higher (3.05 MRayl) than the target and a thickness of 13 μm. With this configuration, the second matching layer can also be used to attach an elevation focusing lens in a future iteration of the device. The backing layer also used the non-conductive PZT-loaded epoxy but at a lower (7% *v*/*v*) fill fraction for an expected impedance of 3.82 MRayl.

**Figure 2:**
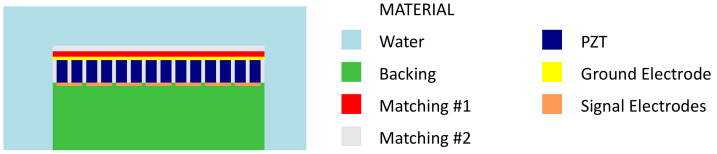
Array geometry used for finite element simulations, where each element is composed of two PZT pillars.

Two-dimensional finite element simulations used the PZT material properties from Lukacs et al., which were measured at 30 MHz. This is near the frequency range of interest and accounts for excimer laser micromachining effects [3,29]. Simulations of the array were performed using the geometry shown in Figure 2 with sub-diced elements; a 2 μm mesh, corresponding to 15 nodes per wavelength; and the material properties in Table 2. The excitation signal was a sinusoidal pulse with a 50 MHz center frequency and 100% frequency bandwidth (−6 dB). The element dimensions and acoustic impedances of the matching and backing layers were varied by as much as 10% to achieve a simulated center frequency of 50 MHz and a fractional bandwidth above 40%, resulting in the dimensions in Table 1. The finite element simulation results are presented in Section 4 and compared with measurements from the fabricated array.

Two highly flexible PCBs are used to route the electrical signals between the driving system and the array elements [32]. The FPCBs are designed with traces at double the element pitch (72 μm) for an interdigitated connection and connected using deposited metals perpendicular to the element electrodes, as shown in Figure 1b,c. The element electrodes are extended with a 100 μm strip of the FPCB at the edge of the array to increase the cross-sectional area for electrical connections. The FPCB traces fan out at the proximal end of the endoscopic device (Figure 1d), here designed to match a 500 μm pitch zero-insertion-force (ZIF) commercial connector (FH52E, Hirose Electric Co., Ltd., Kanagawa, Japan) for interfacing with an imaging system.

## 3. Array Development

### 3.1. Acoustic Stack Fabrication

**Figure 3:**
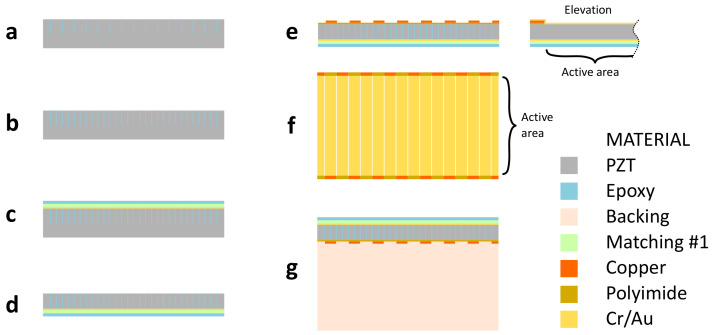
Array fabrication process diagram. (**a**) Azimuth view of dicing and filling of element kerfs in piezocomposite layer. (**b**) Dicing and filling of sub-kerfs. (**c**) Removal of excess epoxy, ground electrode deposition, and casting and lapping of matching layers. (**d**) Lapping of PZT to thickness. (**e**) Addition of FPCB strip and deposition and patterning of element electrodes. Elevation view showing extended electrodes and array’s active area. (**f**) Back surface view of piezocomposite showing laser-patterned signal electrodes and FPCB strips that extend signal electrodes, indicating active area of array. (**g**) Casting and lapping of backing layer. Diagrams not to scale.

The array fabrication process steps are illustrated in Figure 3. To fabricate the piezocomposite, first, a 500 μm thick PZT plate was fixed to a flat carrier. Kerfs separating the elements were ablated at a 36 μm pitch using an ArF excimer laser (193 nm wavelength, 10 ns pulse, IX-255, IPG Photonics, Oxford, MA, USA) with a fluence of 8 J/cm^2^, a 100 Hz pulse repetition frequency (PRF), and a spot size of 3 μm (width) by 90 μm (length). The sample was displaced along the kerf length, with shots spaced by 3.5 μm, executing 20 passes per kerf. Laser ablation parameters were selected following the process described in [32] to achieve an ablation depth greater than the 32 μm target thickness, the narrowest possible kerf, and the highest possible fluence with minimum recast material. The kerfs were cleaned with deionized water, dried with air, and filled with Epo-Tek 301 epoxy, which was cured at room temperature for 24 h (Figure 3a). Sub-kerfs separating elements into two equal pillars were diced and filled with epoxy following the same process. The excess epoxy was removed by lapping with 3 μm alumina until the resulting surface was flat (Figure 3b). A 200 μm wide by 200 μm deep cut was made with a dicing saw (DAD3240, Disco Corporation, Tokyo, Japan) next to the first and last elements to connect the front ground electrode to the back of the array. The ground electrode was deposited by magnetron sputtering (Orion3, AJA International, Scituate, MA, USA) of Cr/Au (300/3000 Å), covering the walls of the 200 μm cuts. The first matching layer was cast, cured at room temperature for 24 h, and lapped to 9 μm thick. Similarly, the second matching layer was cast, cured, and lapped to 13 μm thick (Figure 3c). The epoxy from the second matching layer filled the 200 μm cuts and protected the ground connection to the back. The sample was removed from the carrier and, after cleaning, mounted with the matching layers facing the carrier. PZT was lapped with a 3 μm alumina slurry to obtain a final piezocomposite thickness of 32 μm (Figure 3d). Short, 200 μm long strips of the 72 μm pitch FPCB strips were placed on the lapped piezocomposite at each side of the array elements and aligned to the PZT pillars, with interdigitated even and odd elements (Figure 3e). A thin layer of partially cured Epo-Tek 301 was used to attach the FPCB strips to the piezocomposite, being careful not to cover the array’s active area with epoxy; tweezers were used under a microscope for manual positioning. A signal electrode was deposited by magnetron sputtering of Cr/Au (300/3000 Å) and subsequently removed from alternate filled kerfs using the excimer laser to define the element electrodes (single laser pass, 8 J/cm^2^ fluence, 100 Hz PRF, 3 μm × 90 μm spot size, 3.5 μm shot spacing). The deposited metals were then removed from between the extended electrode traces (single laser pass, 0.5 J/cm^2^ fluence, 100 Hz PRF, 30 μm × 90 μm spot size, 18 μm shot spacing) (Figure 3e).

The electrical impedance of all elements was measured at the extended traces on the PZT composite with a vector network analyzer connected to a micro-probe station to confirm electrode continuity and adhesion. Additional details of electrical impedance measurements are presented in Section 4. The backing layer was cast, cured at room temperature, and lapped to 1.5 mm in thickness (Figure 3g). The acoustic stack was diced to its final dimensions, which exposed the cross-section of the FPCB traces that extend the element electrodes. The electrical impedance of all elements was measured with the micro-probes at the exposed element electrodes.

### 3.2. Flexible-Circuit Fabrication

The copper traces of the FPCB were patterned as illustrated in Figure 1d to form the signal layer. Here, a standard photolithographic approach was used, although we have also demonstrated a laser-patterned FPCB [32]. A copper-on-polyimide film was fixed to a flat carrier, and the copper surface was cleaned with acetone, isopropyl alcohol, and deionized water and dried with nitrogen. A layer of photoresist (Microposit S1818, Dow Chemical Company, Midland, MI, USA) was spin-coated at 4000 RPM to obtain a 2 μm thick layer. The sample was baked at 90 °C for 90 s and exposed to UV light for 40 s to transfer the pattern from a chromium mask. The exposed photoresist was developed using MF-319 (Shipley Co., Marlborough, MA, USA) for 40 s. The exposed copper was etched with ferric chloride for around 4.5 min until the copper was fully removed down to the polyimide layer and all traces were electrically separated from each other. The photoresist was removed with acetone. A ground layer, consisting of a copper-on-polyimide film, and a polyimide coverlay were laminated, leaving 500 μm of copper traces exposed on the array end and 3 mm exposed on the connector end. Finally, the FPCB was cut to its final dimensions.

### 3.3. Array Assembly and Connection

The FPCBs were connected to the array one side at a time. The traces of the first FPCB were aligned to the even array elements and fixed with a small amount of partially cured epoxy loaded with alumina (15% *v*/*v*) approximately 10 min after mixing. We have found that sputtered metals adhere and undergo laser patterning better when the substrate is loaded with particles, in this case, alumina, for the adhesive and PZT for the backing layer. After curing at room temperature for 24 h and then at 65 °C for 2 h, Cr/Cu (300/3000 Å) electrodes were deposited and laser-patterned (single laser pass, 0.5 J/cm^2^ fluence, 100 Hz PRF, 30 μm × 90 μm spot size, 18 μm shot spacing) to separate adjacent element traces. This procedure was repeated for the odd elements on the opposite side of the acoustic stack. The electrical impedance of all elements was measured at the FPCB connector end.

Copper electroplating was performed to improve the electrical connection between the laser-patterned, vacuum-deposited metals and the array elements. The array was placed in a copper acetate solution, and the FPCB traces were connected to 1 V_*DC*_ for 30 s. The electrical impedance of all elements was measured at the FPCB connector end. Finally, the connections were covered with a thin epoxy layer for protection.

The FPCB was connected to a programmable Vevo F2 (Software version 5.7.1.2875) beamforming system (Fujifilm Visualsonics, Toronto, ON, Canada) via an interposer PCB that was compatible with the micro-coaxial cable bundle of the Vevo F2 system. The interposer board also enabled access to individual elements for acoustic characterization tests.

## 4. Array Characterization

### 4.1. Electrical Impedance

The electrical impedance of the array elements was measured to evaluate the electric response of the array at different fabrication stages. A vector network analyzer (E5061B, Agilent Technologies, Santa Clara, CA, USA) was used to measure the complex impedance in the operating frequency bandwidth. Micro-probes were used to make contact with the ∼30 μm wide element electrodes. The effective electromechanical coupling factor, $k_{t}$, was found using

(2) $$k_{t}^{2}=\frac{\pi }{2}\frac{f_{r}}{f_{a}}\tan\left[\frac{\pi }{2}\left(\frac{f_{a}-f_{r}}{f_{a}}\right)\right],$$

where $f_{r}$ and $f_{a}$ are the series and parallel resonant frequencies, respectively [36].

### 4.2. Acoustic Characterization

Pulse-echo measurements were performed with the fabricated array to evaluate the functionality of the array elements. The array was placed in deionized degassed water at different distances from a polished quartz glass. Individual elements were excited with a sinusoidal, 15 V_*pp*_, 50 MHz center frequency pulse and a 100% fractional bandwidth (−6 dB) generated by an arbitrary waveform generator (AWG; PXDAC4800, Signatec, Lockport, IL, USA). Echo waveforms were bandpass-filtered (8–100 MHz) and recorded with an oscilloscope (DSO9064A, Agilent Technologies, Santa Clara, CA, USA).

The amplitude spectrum of the reflected pulse was calculated using a Fast Fourier Transform (FFT) and the bandwidth determined by the two frequencies at which the spectrum dropped by 6 dB from its maximum value. The center frequency was calculated as the mean of the two frequencies. The −6 and −20 dB pulse lengths were determined from the time interval in which the envelope amplitude remained above 1/2 and 1/10 of the peak amplitude, respectively. The peak amplitude was used as a measure of relative sensitivity.

To calculate the insertion loss, individual array elements were excited with a 10-cycle, sinusoidal, 5 V_*pp*_, 50 MHz center frequency pulse generated by the AWG. The echo reflected from the quartz was recorded with the oscilloscope. The recorded signal was divided by the input signal, converted to decibels, and averaged over all elements. These results were compensated for attenuation in water (2.0 × 10^−4^ $f^{2}$ dB/mm-MHz^2^) and the reflection coefficient of the quartz flat (0.82) [37].

The combined acoustic and electrical crosstalk of the array and FPCB was measured by exciting an element and recording the voltage in the four adjacent elements. The array was placed in a deionized degassed water tank with no reflector in front. Element 48 was excited with a sinusoidal, 1.5 V_*pp*_, 50 MHz center frequency 100%, fractional bandwidth pulse. The excitation signal was recorded with the oscilloscope as a reference. The voltage in four adjacent elements (49 to 52) was recorded. In the frequency domain, the spectrum amplitude was normalized by the reference value and converted to decibels. These measurements were repeated, exciting element 20 and recording the voltage in four adjacent elements (21 to 24). From the two sets of measurements, the average crosstalk was computed.

The pressure field was recorded by scanning a 40 μm needle hydrophone (Precision Acoustics, Dorchester, UK) in the azimuthal plane at different distances from the transducer in deionized degassed water. First, the pressure field of a single element was recorded, exciting it with a 15 V_*pp*_, 50 MHz center frequency, 100% fractional bandwidth pulse from the AWG. The element directivity was calculated using the −6 dB beamwidth at a 5 mm distance. Then, the pressure field of beams focused at 5 and 7 mm depths and on different numbers of active elements was measured. Between 32 and 64 elements were driven with the Vevo F2 beamformer with a single-cycle, 15 V_*pp*_, 50 MHz center frequency excitation signal.

### 4.3. Laser Doppler Vibrometry

Laser Doppler vibrometry measurements were made to evaluate the performance of the acoustic stack in air and compare it with the simulation results. The vertical displacement of the top surface of the transducer in air was measured with a UHF-120 (Polytec GmbH, Waldbronn, Germany) after connecting the acoustic stack to the FPCB, addressing individual elements via the PCB. Array elements were excited one at a time with a single-cycle sinusoidal, 15 V_*pp*_, 50 MHz center frequency pulse. Time-based displacement measurements were acquired for 500 ns with a sampling period of 1 ns. Measurements were spaced by 2 μm in the azimuth and 200 μm in the elevation directions. The envelope of the displacement was calculated using the Hilbert transform and converted to a dB scale.

### 4.4. Imaging

**Figure 4:**
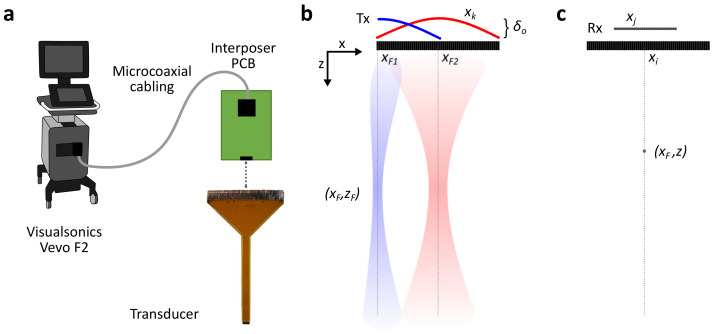
The imaging setup and beamforming. (**a**) An array connected to the imaging system via micro-coaxial cabling and the interposer PCB. (**b**) Transmit beamforming showing delay profiles for an aperture at the center of the array and another toward one edge, resulting in a steered focused beam. (**c**) Receive beamforming for an image line at $x_{F}$ using a receive aperture of elements at $x_{j}$.

Imaging tests were performed to evaluate the array functionality. The transducer was connected to a Vevo F2 system via the PCB and micro-coaxial cabling (Figure 4a), and B-mode images of different targets were obtained. Line-by-line imaging was performed in the Vevo Advanced Data Acquisition (VADA) mode, transmitting on up to 64 elements and receiving on up to 32 (beamformer channel count limit), and exported for offline beamforming and reconstruction. Transducer elements were excited with a single-cycle sinusoidal, 15 V_*pp*_, 50 MHz center frequency pulse.

The image was reconstructed using delay-and-sum beamforming [38,39]. Referring to the array geometry in Figure 4b and assuming a uniform speed of sound *c*, the time of arrival (TOA) of a pulse transmitted by an element ($x_{k}$,0) to a point (*x*,*z*) can be written in terms of the time of flight (TOF) and an arbitrary delay *δ* as

(3) $$TOA^{Tx}\left(x,z;x_{k}\right)=TOF\left(x,z;x_{k}\right)+\delta ,$$

with

(4) $$TOF\left(x,z;x_{k}\right)=\frac{1}{c}\sqrt{{\left(x_{k}-x\right)}^{2}+z^{2}}.$$

The beam can be focused by choosing a delay for each element such that the pulses from all active elements arrive in phase at a focal point ($x_{F}$,$z_{F}$) and coherently compound. The delay for element *k* can be written as

(5) $$\delta \left(x_{F},z_{F};x_{k}\right)=TOF\left(x_{F},z_{F};x_{k}^{dist}\right)-TOF\left(x_{F},z_{F};x_{k}\right),$$

where $x_{k}^{dist}$ is the most distal element from $x_{F}$. The transmit time of arrival to any point along the imaging line can be approximated as the sum of the transmit time of a plane wave propagating from the transmit aperture and the transmit delay of the element at $x_{k}^{prox}$:

(6) $$TOA^{Tx}\left(x_{F},z\right)\approx \frac{z}{c}+\delta \left(x_{F},z_{F};x_{k}^{prox}\right),$$

where $x_{k}^{prox}$ is the element closest to the imaging line, which can fall between two elements of the transmit aperture.

Figure 4c shows the receive aperture for an image line at $x_{F}$, which, in general, can be different from the transmit aperture. The time of flight from a scatterer at ($x_{F}$,*z*) to the receive element at $x_{j}$ can be calculated with Equation (4), namely, $TOF(x_{F},z;x_{j})$. The total time for the signal to travel from the transducer to the scatterer at ($x_{F}$,*z*) and back to the receive element at position $x_{j}$ is

(7) $$\tau \left(x_{F},z;x_{j}\right)=TOA^{Tx}\left(x_{F},z\right)+TOF\left(x_{F},z;x_{j}\right)\approx \frac{z}{c}+\delta \left(x_{F},z_{F};x_{k}^{prox}\right)+TOF\left(x_{F},z;x_{j}\right).$$

A beamformed signal $S_{BF}\left(x_{F},z\right)$ can be calculated at any point along the image line by coherently compounding the radiofrequency (RF) signal $S_{j}$ collected by each element in the receive aperture delayed by *τ*, where *τ* is equal to the time delay calculated above for each element in the receive aperture:

(8) $$S_{BF}\left(x_{F},z\right)=\sum_{j=1}^{N}S_{j}\left[\tau_{j}\left(x_{F},z;x_{j}\right)\right].$$

The imaging line was shifted by choosing a new transmit aperture and delay profile, and the same beamforming process was performed. The number of active elements was reduced toward the array edges, resulting in a steered focused beam. Figure 4b shows two beams centered at different image lines $x_{i1}$ and $x_{i2}$ and the corresponding transmit delays for each case.

For our imaging experiments, a transmit f-number of 2.5 was chosen to use the full aperture when focusing at around 5 mm. Dynamic receive beamforming with a constant f-number of 2.0 was used, such that the number of receive elements used for beamforming increased with the imaging depth up to 32 elements. The number of active elements on receive was reduced toward the edges of the FOV. The envelope of the beamformed RF data was calculated, log-compressed, and displayed in grayscale. The signal-to-noise ratio (SNR) was measured using

(9) $$\mathrm{SNR}=20\log\left(\frac{E_{S}}{E_{N}}\right),$$

where $E_{S}$ and $E_{N}$ are the means of the signal envelope from a region of interest (ROI) corresponding to tissue and background noise, respectively. ROIs were selected at similar depths.

#### 4.4.1. Wire Phantom

A fine-wire target was imaged to measure the axial and lateral resolutions of the array. Images of an 8 μm diameter tungsten wire (Midwest Tungsten Service, Willowbrook, IL, USA) aligned with the array elevation were obtained in deionized degassed water, positioning the wire at different depths and lateral positions. The resolution was calculated by measuring the full width at half maximum (FWHM) of the axial and lateral line spread functions.

#### 4.4.2. Ex Vivo Porcine Esophageal Tissue Imaging

Esophageal tissue was obtained ex vivo from a 67 kg Yorkshire swine that had been utilized for a separate experiment. The specimen was cut in the longitudinal direction to expose and image the luminal side. The array was coupled acoustically with a small amount of US gel. For comparison, the specimen was also imaged with a commercial array that operates in the 30 to 71 MHz bandwidth (UHF71x, Fujifilm Visualsonics) and has an image width up to 9.7 mm. The image width was limited to 2 mm, equal to the field of view of the fabricated array. The transmit power was chosen to avoid saturating the amplifiers with the signal from the first echogenic interface. The same imaging sequence and beamforming process were used for both arrays.

Following US imaging, selected porcine esophageal samples were sectioned and fixed in 10% formalin solution at room temperature for 24 h. The samples were dehydrated, embedded in paraffin, sectioned in 5 μm thick slices with a microtome, and mounted on glass slides. Hematoxylin and eosin (H&E) staining was performed, and the samples were examined under a light microscope.

#### 4.4.3. In Vivo Imaging

A fertilized duck egg was transferred to a plastic weigh boat on the fourth day of embryonic development (EDD-4). On EDD-12, the embryo was imaged in real time with our transducer using a small amount of phosphate-buffered solution (PBS) for coupling. The field of view was 10.0 mm (axial) by 2.3 mm (lateral). Images of superficial vessels and of the beating heart were obtained in different orientations.

## 5. Results

### 5.1. Array Fabrication

**Figure 5:**
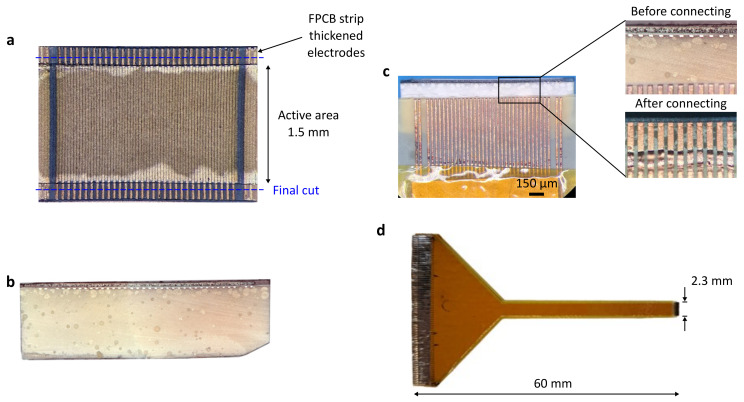
The array transducer at different fabrication stages. (**a**) A view of the back surface of the piezoelectric layer after adding FPCBs to extend the element electrodes and depositing and patterning the signal electrode and before adding the backing layer. (**b**) The side view of the diced and patterned array showing the exposed electrode cross-section of FPCB extensions. (**c**) The array aligned to FPCB traces before and after metal deposition, patterning, and electroplating. (**d**) The array connected to the FPCB with fan-out to 500 μm pitch pads compatible with a commercial zero-insertion-force connector.

Photographs of the array at different fabrication stages are shown in Figure 5. The 2-2 piezocomposite with patterned Cr-Au array electrodes and FPCB strips for extending the electrodes is shown before casting the backing layer and trimming to the final dimensions (Figure 5a). The FPCB strips are aligned to alternate elements, interdigitated over the active area. The FPCB traces are 8 μm thick and therefore have a much larger cross-sectional area than the deposited Cr-Au electrodes. The positions of the dicing saw cuts are indicated; only 50 μm of the extended electrodes remain as part of the transducer. One edge of the array after adding the backing layer and dicing it to its final dimensions is shown in Figure 5b. Figure 5c shows the FPCB aligned to the even electrodes of the array before and after metal deposition, laser patterning, and electroplating. The physical appearance of the traces did not change considerably after electroplating. The array connected to the FPCB and ready for characterization tests is shown in Figure 5d. The two FPCBs add 100 μm to the elevation, which, in addition to the extended electrodes, corresponds to a total of 200 μm added to the transducer footprint.

### 5.2. Electrical Impedance

**Figure 6:**
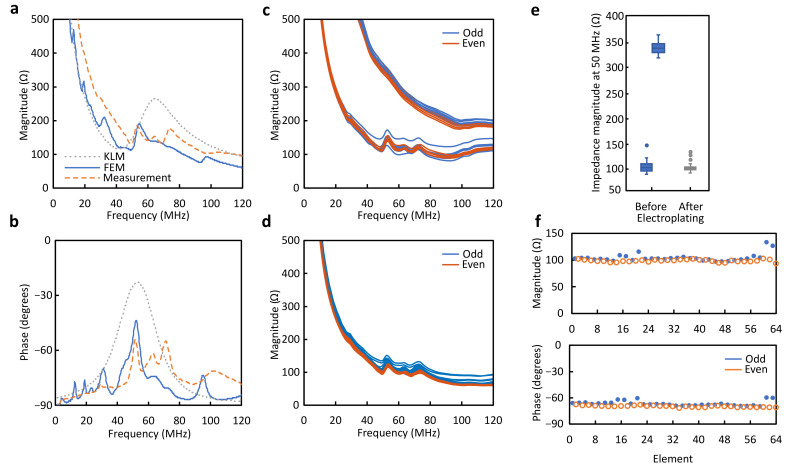
Electrical impedance measurements during fabrication. (**a**) Impedance magnitude and (**b**) phase of representative element measured at edge of element electrode, compared with FEM and KLM simulation results. (**c**) Impedance measured at connector end of FPCB before and (**d**) after electroplating indicating odd and even elements, connected on opposite sides. (**e**) Comparison of electroplating effect on impedance magnitude at 50 MHz in all elements. (**f**) Element impedance uniformity at 50 MHz measured at FPCB.

The electrical impedance of individual elements was measured after the backing layer was added and the array was diced to expose the element electrodes, such as in Figure 5b. The measured electrical impedance magnitude and phase from one representative element (orange dashed line) are shown in Figure 6a,b, along with the KLM (gray dotted line) and FEM (blue solid line) simulation results. Measurements from all elements display an average electrical impedance magnitude of 136.9 ± 6.06 Ω and a phase of −63.92 ± 3.67° at 49.2 MHz. A relatively low standard deviation in impedance magnitude values indicates a uniform piezoelectric response across the array and successful connection to the extended electrodes. An electromechanical coupling coefficient of 44.3 ± 7.65% was calculated using Equation (2).

The electrical impedance magnitude of all elements measured at the FPCB connector end is shown before (Figure 6c) and after (Figure 6d) electroplating, indicating odd and even elements. Figure 6c displays two separate groups of curves, plotted independently in Figure 6e, indicating that 18 of the 64 elements, with a higher impedance magnitude value and without resonance, were not properly connected to their corresponding traces on the FPCB. Electroplating improves the uniformity of the electrical impedance at resonance (Figure 6e) and in the entire frequency bandwidth (Figure 6d). Element uniformity plots (Figure 6f) show that all elements were connected to the FPCB, with an average impedance magnitude of 102.08 ± 6.36 Ω and a phase of −67.87 ± 2.64° at 49.2 MHz. Adding the FPCB reduces the average electrical impedance magnitude value by 25% but has no effect on the resonant frequency.

### 5.3. Acoustic Characterization

**Figure 7:**
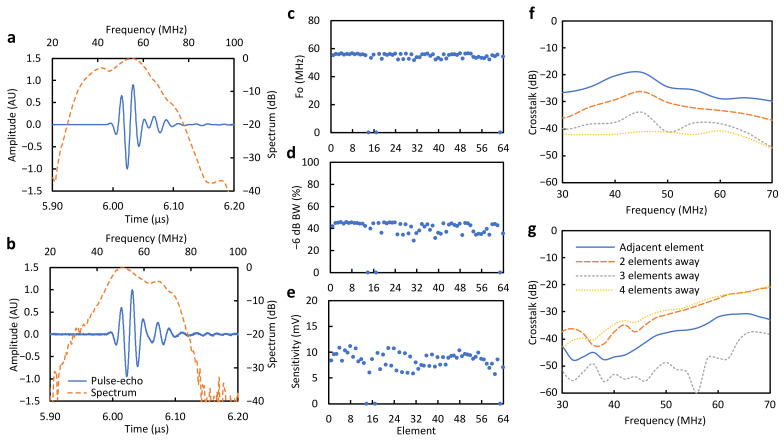
Pulse-echo time series (solid line) and frequency spectrum (dashed line) from (**a**) FEM simulation and (**b**) experiment of representative element (#48). Element uniformity of (**c**) center frequency, (**d**) fractional bandwidth (−6 dB), and (**e**) sensitivity from pulse-echo measurements. Combined electrical and acoustic crosstalk from (**f**) FEM simulation and (**g**) experiment. pressure field from hydrophone measurements.

The simulated pulse-echo response for the transducer is shown in Figure 7a. Simulations yielded a −20 dB pulse length of 87.1 ns and 59% −6 dB bandwidth about a center frequency of 49 MHz. The measured pulse from element #48 has a similar shape and slightly longer ringdown (Figure 7b). The average measured −6 dB and −20 dB pulse lengths were 32.4 ± 0.5 and 93.0 ± 3.0 ns, respectively, 4% and 6.7% longer than the simulated pulse length. The average measured center frequency of 54.9 ± 1.5 MHz is 11% higher than simulated. The measured −6 dB and −20 dB frequency ranges were determined to be 43.6 to 66.1 MHz and 30.5 to 78.8 MHz, respectively. In contrast, simulations indicated a wider range at −6 dB, from 34.6 to 63.5 MHz. The pulse-echo responses, particularly bandwidth and sensitivity, had less uniformity across elements compared to electrical impedance measurements. Three open elements were found in this prototype, which is comparable to other micro-US arrays reported in the literature [23].

An average insertion loss of −29.75 ± 1.47 dB was calculated after compensating for attenuation in water and the reflection loss from the quartz target. This value is higher than comparable values reported for arrays operating at slightly lower frequencies (35 MHz), with larger array elements and better electrical matching to the driving system [5,40]. However, it is an improvement to miniaturized arrays with similar operating frequencies and dimensions [21,23].

The simulated and measured crosstalk are shown in Figure 7f,g, respectively. The measured crosstalk level at a 50 MHz center frequency was below −29 dB for all four adjacent elements, whereas, from simulations, a crosstalk below −24 dB was expected. Simulated crosstalk generally decreases with frequency across the device bandwidth and has a lower absolute value as the distance from the excited element increases. On the contrary, measured crosstalk increases overall with frequency and is significantly higher for distances at two and four elements away, with a difference of about 8 dB for frequencies above 55 MHz. This coupling is expected, particularly between neighboring traces running on the same FPCB layer.

**Figure 8:**
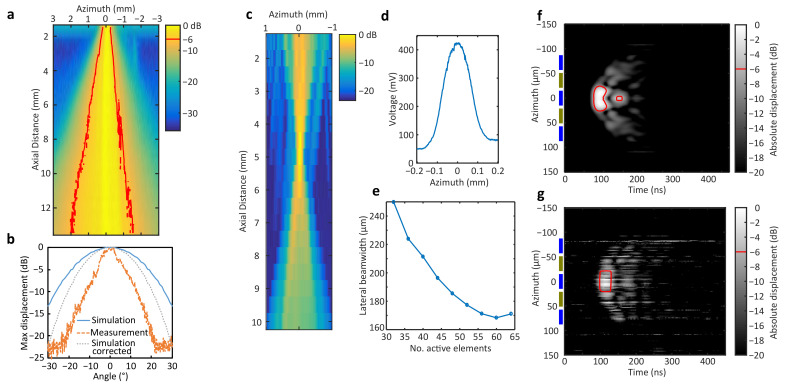
Pressure field and vibrometry measurements. (**a**) Element pressure field from hydrophone measurements, showing −6 dB contour in red. (**b**) Element directivity from simulation (solid blue line), hydrophone measurements (dashed orange line), and simulation corrected for hydrophone directivity (dotted gray line). (**c**) Measured pressure field, (**d**) beam profile at focus for beam focused at 5 mm using 60 elements, and (**e**) beamwidth (−6 dB) variation with number of active elements. Surface displacement envelope from (**f**) FEM simulations and (**g**) vibrometry measurements for single active element, showing −6 dB contour and relative position of array elements connected to the same (blue) and opposite (olive) sides of array.

The element pressure field in the azimuth plane from hydrophone measurements is shown in Figure 8a, displaying the −6 dB contour line. Figure 8b shows the simulated (solid line) and measured (dashed line) one-way element directivities obtained from hydrophone measurements. Because the hydrophone size is about one wavelength, its acceptance angle should be considered. Therefore, the simulated directivity corrected for the hydrophone directivity is shown for comparison.

The pressure field of the beam focused at 5 mm has a −6 dB beamwidth of 168 μm (Figure 8c,d) when using 60 active elements. This is the narrowest beamwidth achieved, which suggests that, because of the narrow element directivity, increasing the active aperture beyond 60 elements degrades the focusing performance (Figure 8e).

### 5.4. Laser Doppler Vibrometry

The envelope of the surface displacement for simulations is shown in Figure 8f. The −6 dB contour line is displayed and has a width of 40 μm. The envelope of the displacement time series obtained from laser vibrometry measurements is shown in Figure 8g. Similarly, the −6 dB contour has a 40 μm width and a shape similar to that in simulations. A margin of error is expected due to the 10 μm laser spot size of the vibrometer. Five elements are shown next to the vertical axis for reference, where alternate elements (same color) are connected to traces in the same FPCB. A different surface wave speed of sound between simulations and measurements is evident from the angle subtended by the wavefront.

### 5.5. Imaging

**Figure 9:**
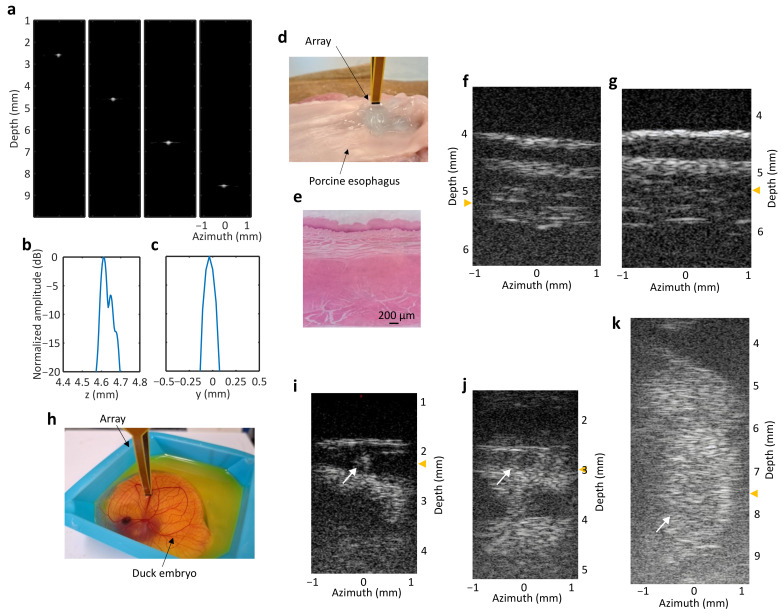
Fine wire, ex vivo, and in vivo imaging. (**a**) Images of 8 μm wire at different depths with 40 dB dynamic range; (**b**) axial (−43 dB noise floor) and (**c**) lateral resolutions (−30 dB noise floor) for wire at 4.5 mm depth. Ex vivo imaging of porcine esophagus: (**d**) imaging setup, (**e**) hematoxylin-and-eosin-stained histology section, and images acquired with (**f**) fabricated array and (**g**) commercial 50 MHz array and reconstructed offline. Chorioallantoic membrane model imaging (**h**) setup and images showing superficial vessel in (**i**) transverse and (**j**) longitudinal planes and (**k**) heart. Marker: focal depth.

Images of the fine wire are shown in Figure 9a for 2.5, 4.5, 6.5, and 8.5 mm depths in a 40 dB dynamic range. The measured axial and lateral resolutions at 4.5 mm were 35 μm and 114 μm, respectively (Figure 9b,c). The lateral resolution worsens with depth because of the limited aperture. For the commercial array, we obtained axial and lateral resolutions at 4.5 mm of 39 μm and 120 μm, respectively, using the same signal acquisition and data processing as the fabricated array.

Ex vivo images of a porcine esophagus are shown in Figure 9d–g. The experimental setup shows the transducer orientation with respect to the open esophagus (Figure 9d), and the image obtained with the fabricated transducer is displayed in Figure 9f in a 40 dB dynamic range. An SNR of 12 dB was obtained between the signal in the top layer of tissue compared to the gel above it. Different echogenic layers can be correlated with histology (Figure 9e), which shows a 2 mm thick section of tissue. An image with comparable detail was obtained with the 50 MHz commercial array, shown in Figure 9g, with the field of view reduced to match that of the fabricated array. An SNR of 18 dB was calculated.

Duck embryo images obtained in vivo with the fabricated transducer are shown in Figure 9h–k. The transducer positioned over the ex ovo embryo is shown in Figure 9h. Images of a superficial vessel are displayed in Figure 9i,j and indicated with a white arrow in the transverse and longitudinal planes, respectively. An average SNR of 9 dB was calculated. Figure 9k shows an image of the beating heart, indicated with a white arrow. A video of the beating heart is available in Supplementary Materials. The focal depth is indicated with yellow arrowheads on the vertical axis in each image.

## 6. Discussion

Reducing the space occupied by the connection between the array and cabling is a significant challenge for fabricating endoscopic or miniature arrays. The method presented here allows a more compact integration of the array and interconnect compared to the cumbersome bending of the FPCB toward the back or side of the array, as implemented in previous work [22,23,24,25,26]. Vacuum-deposited metals are widely used as electrodes in piezoceramic and MUT transducers [17,20]. Laser micromachining has also been used to pattern element electrodes on piezoceramic substrates [41,42]. In [32], we used vacuum-deposited metals and laser patterning to connect array elements at the front of an array. However, to our knowledge, this is the first array that combines the two processes plus electroplating to achieve a right-angle connection between signal electrodes at the back of the array and the FPCB traces, allowing cast quarter-wavelength matching layers to be used. Thickened electrodes at the array edge are important to increase their cross-sectional area and facilitate connection. Attempts to thicken the back-face electrodes at their edges by electroplating did not result in a uniform thickness, straight edges, or electrical continuity. Our solution to use short strips of patterned FPCBs on each array end provided a reliable electrical connection and uniform copper traces to connect to. Additionally, the electroplating step after metal deposition and laser patterning to create the connection between the extended array electrodes and the FPCB was sufficient to restore the open connections resulting from the laser patterning of the thin-film metals. The impedance magnitude (at 50 MHz) of a subset of the elements was about four times higher than the rest of the elements immediately after laser patterning and had no apparent piezoelectric resonance. This is expected of a poor or open connection between the signal electrodes on the array and the FPCB traces. Upon inspection, we determined that the increased impedance was due to metal delamination caused by laser machining. Several unsuccessful combinations of backing layer substrates, laser parameters, and metal deposition processes were tested to improve metal adhesion to the substrate and avoid connection failure. We found that, to achieve an acceptable fabrication yield, it is important to utilize an epoxy substrate loaded with particles such as aluminum oxide or PZT, a plasma etching step before metal deposition, low laser fluence during laser patterning, and a final copper electroplating procedure that restores the connection of discontinuous traces. This last step also improves the overall conductivity by around 9%, as evidenced in Figure 6c,d, with a downshift in mean electrical impedance at resonances from 113 to 102 Ω.

Small-footprint devices like the one fabricated have various possible applications that demand high-resolution imaging. The device dimensions make it suitable for packaging in an 11F (3.66 mm outer diameter) catheter, which could be useful for endoscopic imaging applications. Superficial or intraoperative imaging can be achieved with short compact arrays. Preclinical applications that require a small-footprint device, such as in ovo avian embryonic imaging, can be achieved with such compact devices that fit in the 1–2 cm access hole in the shell [7,8]. Long devices for gastrointestinal procedures can be achieved by transitioning from the FPCB to a micro-coaxial cable bundle next to the array, which makes them flexible enough to be easily navigated. The fabrication approach presented can also be used to manufacture phased arrays, which allows the steering of the acoustic beam to give a broad field of view from a small aperture [27]. This approach would require an element pitch of about a half-wavelength to avoid grating lobes and may be feasible using fine-pitch FPCBs previously reported [32].

We were able to image swine tissue and avian embryos to a depth of around 10 mm. The images acquired with the fabricated probe showed similar layering of the esophageal wall to those acquired with the commercial probe, but with a lower signal-to-noise ratio. It should be noted that the built-in imaging modes of the Vevo F2 system have qualitatively better images than the reconstruction used here for the control of array elements and direct comparisons without the extensive optimization of signal processing. The image resolution degrades toward the edges due to the use of fewer active elements, which becomes a limitation for linear arrays with relatively short apertures. The number of elements for a forward-looking array is restricted by the maximum size allowed by the package. However, in a side-looking array, which would be more useful for certain applications (e.g., imaging the gastrointestinal wall), it is possible to increase the number of elements and therefore the imaging aperture. The slice thickness could be improved by implementing an elevation focusing lens. The second unloaded epoxy matching layer is suitable for attaching a polymethylpentene lens, which has an acoustic impedance similar to that of tissue [3,5].

After performing a set of acoustic characterization experiments, the connection from the FPCB to three elements failed. Electroplating for longer than 30 s could prevent open elements by producing thicker and wider copper traces able to carry larger currents. Additionally, electrical impedance matching could be implemented at the PCB to match the 50 Ω characteristic impedance of the driving system, improving sensitivity.

The higher-than-expected center frequency can be attributed to a deviation from the designed active layer thickness, which can be challenging to control to a micrometer accuracy for a piezocomposite using a soft epoxy [43], or to differences in simulated and actual material properties. When making the composites for the matching and backing layers, it is also difficult to ensure a uniform distribution of the loading powder and, therefore, the accurate control of the material properties of the layer [44], which could result in bandwidth and sensitivity variations. The higher center frequency of the fabricated array also explains its slightly better axial and lateral resolutions than the commercial transducer.

Crosstalk is a source of image quality degradation and deserves further investigation. The measured crosstalk for the adjacent element is lower than predicted by simulations. Conversely, crosstalk for elements at two and four elements away is higher than expected from simulations. Simulations do not account for the electrical load from the FPCB or electrical crosstalk between FPCB traces, which need to be considered before making a direct comparison between simulations and measurements. Crosstalk between elements connected to traces running on the same FPCB could be reduced with additional shielding. However, vibrometry measurements (Figure 8g) show no surface displacement for these elements at 100 ns, the time at which signals from the excited elements (at 0 μm azimuth) appear. Therefore, the electrical crosstalk between neighboring traces on the same FPCB (colored in blue) is not sufficient to induce a simultaneous displacement in the inactive elements.

Determining the element directivity directly from hydrophone measurements is limited due to the hydrophone acceptance angle. In our experiments, the hydrophone was scanned only by translation without re-orientation in the direction of the element. The 40 μm hydrophone membrane is on the order of one wavelength, and therefore, its sensitivity falls with the increasing angle from the axis direction. This partly explains the deviation of our results from the theoretical directivity (−6 dB) of around ±20°, which would be expected for an element 2.5 times wider. Vibrometry measurements confirm surface displacement within 6 dB of the peak displacement from a region 1.3 times wider than the element. The speed of lateral wave propagation within the array, as shown by the time of the initial surface displacement along the azimuth, is faster in the vibrometry measurement than in the simulation, which may contribute to a larger effective element width. Element directivity could be improved by dicing through the matching layers to reduce the acoustic cross-coupling between elements [27] or by optimizing the materials and dimensions of the piezocomposite substrate [45].

## 7. Conclusions

We have demonstrated the fabrication of a 2.3 mm by 1.7 mm, 64-element linear array with elements at a 36 μm pitch connected to a 4 cm long FPCB. We presented a method that achieves a right-angle connection between the array and the FPCB, eliminating the need for a bending radius and adding only 200 μm overall to the array elevation. Element electrodes were thickened at the edge by connecting short, patterned circuit traces to the electrodes on the back surface of the piezocomposite in an interdigitated manner. The array was connected to FPCBs with traces at a 72 μm pitch using vacuum-deposited, laser-patterned, and electroplated copper. Electroplating restored open element connections, resulting in an improved yield and reliability. The average element center frequency of the fabricated array was measured at 55 MHz, with a −6 dB fractional bandwidth of 41% and a combined crosstalk of less than 29 dB. The axial and lateral image resolutions measured at 4.5 mm with an 8 μm diameter tungsten wire were 35 μm and 114 μm, respectively. The array was connected to a commercial ultrasound system, and imaging was successfully demonstrated by visualizing the layered structure of porcine esophageal tissue ex vivo and an avian embryonic heart and CAM microvasculature in vivo.

## Figures and Tables

**Table 1:** Transducer specifications.

Parameter	Value
Number of elements	64
Pitch	36 µm
Element width (azimuth)	30 µm
Kerf width	6 µm
Element length (elevation)	1.5 mm
Element thickness	32 µm
Matching 1 thickness (PZT–epoxy)	9 µm
Matching 2 thickness (epoxy)	13 µm
Backing thickness (PZT–epoxy)	1.5 mm

**Table 2:** Material properties used for 2-D finite element simulations.

Parameter	Symbol	Epoxy ^a^	PZT ^b^	PZT (7%)–Epoxy ^c^	PZT (32%)–Epoxy ^c^
Density (kg/m^3^)	ρ	1150	7820	1583	3589
Longitudinal velocity (m/s)	v_L_	2650	4550	2425	2156
Shear velocity (m/s)	v_S_	1230	2275	1140	1098
Acoustic impedance (MRayl)	Z	3.05	35.6	3.83	7.74
Attenuation (dB/mm)	α	9.5	1.71	42	29
Dielectric constant	$\varepsilon_{11}^{T}$		2417		
	$\varepsilon_{33}^{T}$		3000		
Piezoelectric charge constant (pC/N)	d_15_		560		
	d_31_		−295		
	d_33_		481		

^a^ Epotek 301. Epoxy Technology, Inc., Billerica, MA, USA [35]. ^b^ 3203HD. CTS Corporation, Lisle, IL, USA [3]. ^c^ Epotek 301 + PZT powder. Estimated using [34,35].

## Data Availability

Data supporting the reported results are available upon request.

## Supplementary Materials

The following supporting information can be downloaded at: https://www.mdpi.com/article/10.3390/s24061847/s1, Video S1: Avian embryonic heart imaged with the fabricated array.

## Abbreviations

The following abbreviations are used in this manuscript:

ASIC	Application-specific integrated circuit
AWG	Arbitrary waveform generator
CAM	Chorioallantoic membrane
FEM	Finite element model
FFT	Fast Fourier Transform
H&E	Hematoxylin and eosin
FOV	Field of view
FPCB	Flexible printed circuit board
MUT	Micromachined ultrasound transducer
PCB	Printed circuit board
PZT	Lead zirconate titanate
RF	Radiofrequency
ROI	Region of interest
SNR	Signal-to-noise ratio
TOA	Time of arrival
TOF	Time of flight
US	Ultrasound
ZIF	Zero insertion force
